# Integration across Time Determines Path Deviation Discrimination for Moving Objects

**DOI:** 10.1371/journal.pone.0001930

**Published:** 2008-04-16

**Authors:** David Whitaker, Dennis M. Levi, Graeme J. Kennedy

**Affiliations:** 1 Department of Optometry, University of Bradford, Bradford, United Kingdom; 2 School of Optometry and Helen Wills Neuroscience Institute, University of California, Berkeley, California, United States of America; University of Minnesota, United States of America

## Abstract

**Background:**

Human vision is vital in determining our interaction with the outside world. In this study we characterize our ability to judge changes in the direction of motion of objects–a common task which can allow us either to intercept moving objects, or else avoid them if they pose a threat.

**Methodology/Principal Findings:**

Observers were presented with objects which moved across a computer monitor on a linear path until the midline, at which point they changed their direction of motion, and observers were required to judge the direction of change. In keeping with the variety of objects we encounter in the real world, we varied characteristics of the moving stimuli such as velocity, extent of motion path and the object size. Furthermore, we compared performance for moving objects with the ability of observers to detect a deviation in a line which formed the static trace of the motion path, since it has been suggested that a form of static memory trace may form the basis for these types of judgment. The static line judgments were well described by a ‘scale invariant’ model in which any two stimuli which possess the same two-dimensional geometry (length/width) result in the same level of performance. Performance for the moving objects was entirely different. Irrespective of the path length, object size or velocity of motion, path deviation thresholds depended simply upon the duration of the motion path in seconds.

**Conclusions/Significance:**

Human vision has long been known to integrate information across space in order to solve spatial tasks such as judgment of orientation or position. Here we demonstrate an intriguing mechanism which integrates direction information across time in order to optimize the judgment of path deviation for moving objects.

## Introduction

Newton's first law of motion states that objects in motion tend to stay in motion with the same speed and in the same direction unless acted upon by an unbalanced force. Any deviation or acceleration of a moving object is therefore indicative of some external event having taken place. It is often necessary for humans to use their sense of vision to signal such events and to make revised motor actions in order to either intercept the moving object or else to avoid it, should its new direction pose a threat. Given the importance of detecting deviations in the path of moving objects, it is surprising that the mechanisms involved in this task are poorly understood.

Evidence does exist to suggest that our sensitivity to path deviation should be fairly high. For example, Westheimer and Wehrhahn [1] investigated the ability to discriminate differences in the direction of a moving dot from one presentation to the next. Differences of less than 1° could be reliably discriminated, a value which is close to that obtained for the discrimination of orientation for continuous lines. This raises the intriguing question of whether judgments about paths of motion are based upon some form of static memory trace (or ‘motion smear’ [2]–[5]), upon which subsequent spatial discriminations can be judged.

Movie S1 depicts the type of moving object which forms the basis for the judgments which we measured. An object (a Gaussian blob of a certain size) begins its motion path to the left of the midline (defined by static vertical lines at the centre of the figure). It moves rightwards on a linear path with a certain velocity until the midline, at which stage it suddenly changes its direction, either upwards or downwards. Tripathy and Barrett [6], [7] have used a similar paradigm, but with emphasis upon the tracking of multiple dots, where observers were required to identify the direction of deviation of a single target dot among several non-deviating distracters. In one experiment, Tripathy and Barrett [7] examined thresholds for path deviation of a single dot and observed thresholds of approximately 2°, provided dot velocity was high. Several studies have examined path deviation in terms of reaction time (RT, [8]–10). Observers are presented with a moving object which undergoes a suprathreshold deviation in path direction and observers are required to make a simple or choice reaction to the event. For example, Hohnsbein and Mateeff [9] found that RTs reduced both with the angle of direction change and increasing stimulus velocity.

**Figure 1:**
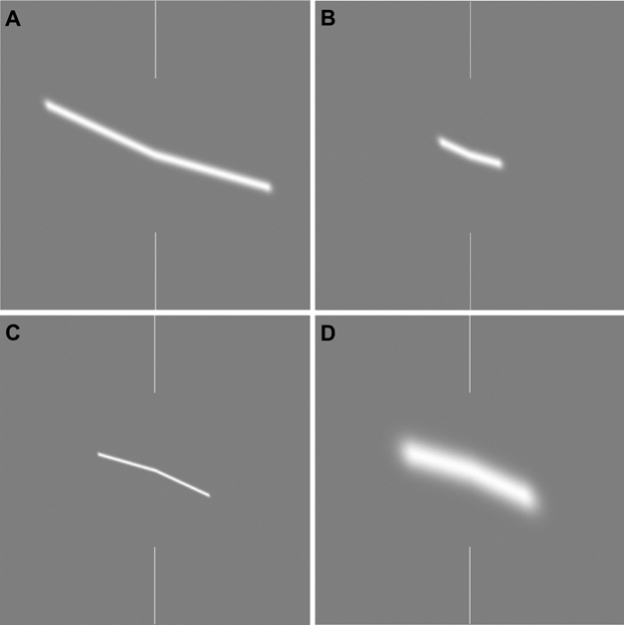
Examples of stimuli. In Experiment 1, stimuli were straight lines with a deviation or ‘kink’ at their midpoint (indicated by white vertical markers). Observers had to indicate the direction of deviation as either ‘upwards’ (panels A and B) or ‘downwards’ (C and D). The parameters that were varied were line length and blur. In Experiments 2 and 3, stimuli were ‘blobs’, which moved rightwards on a linear path with a certain velocity until the midline, at which stage a deviation in the trajectory occurred. Observers again had to signal the direction of deviation. The lines in panels A-D can be considered as a schematic representation of the path traced out by the moving blobs. For the moving stimuli, path length, stimulus blur and viewing distance were varied.

This type of task lends itself to the consideration of several parameters, all of which correspond to the variations in the characteristics of moving objects we encounter in the real world. Objects vary in size and velocity, both as a result of their physical properties and their distance from the observer. The extent of the path of a moving object is also variable, highlighting the fact that a two-dimensional analysis of the world is the problem faced by the visual system in this type of task [11]. We avoided the third dimension by restricting our investigation to path deviations in the fronto-parallel plane. Furthermore, observers may not always have the luxury of a lengthy, unhurried period during which pre- and post-deviation directions can be evaluated. In many sports, in particular, performance depends upon a rapid assessment of direction change in order to make successful motor adjustments. Figure 1 (A–D) depicts the static traces of a variety of object sizes and motion paths investigated in the current study.

We attempted to characterize the mechanisms involved in the detection of path deviation of moving objects by systematically examining the effects of stimulus size, velocity and path length. We also compared performance for moving objects with that for a static trace of the motion path, and this has allowed us to answer the question of whether similar mechanisms are involved in both tasks.

## Results

### Experiment 1

**Figure 2:**
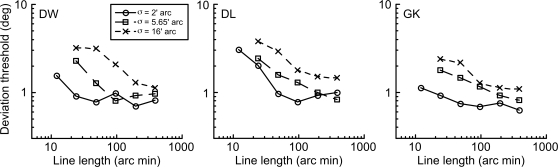
Angular discrimination thresholds for static lines. Thresholds for detecting deviations in straight lines, such as those in Figure 1, are plotted against line length. Each panel shows data for an individual observer and each symbol type represents a different level of line blur (σ = 2', circles; σ = 5.65', squares; σ = 16', crosses). For the lowest blur level, performance improves with increasing line length and reaches a plateau of approximately 1°. As blur level increases, performance deteriorates for short line lengths but, as line length increases, thresholds improve to approach the threshold plateau for the lowest blur level. This suggests that, provided the line length is sufficiently long, thresholds are independent of blur level. This suggests a process of ‘scale invariance’, in which stimuli which are magnified versions of one another yield identical levels of performance.

We begin by presenting data for the static stimuli in order to provide a baseline data set against which path deviation thresholds for the moving blobs can be compared. Figure 2 shows thresholds for detecting a ‘kink’ in lines such as those presented in Figure 1 (A–D) as a function of the overall length of the line. Data are presented for three levels of blur (σ = 2', 5.65' or 16'arc).

**Figure 3:**
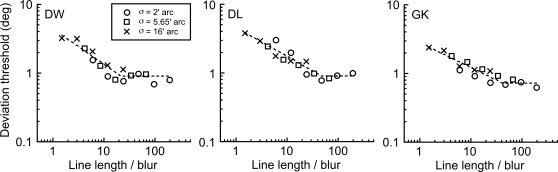
Scale invariance in detecting deviations in static lines. The data from Figure 2 are replotted on a ‘scale invariant’ abscissa, namely line length divided by blur. This collapses together the data at different blur levels and indicates that any two stimuli with identical geometry (same ratio of line length/blur) will produce the same level of performance, irrespective of the absolute size of the stimulus. For each observer, data are fitted with a bilinear function. These line fits indicate that the task of discriminating a deviation from linearity in a straight line is well described by a scale invariant mechanism in which performance reaches a plateau once line length exceeds approximately 40 times the level of blur of the line (the ‘knee’ point).

For the lowest blur level (solid lines–circle symbols), performance improves with line length but soon reaches a plateau at just under 1°. As blur level increases, performance deteriorates markedly at short line lengths but, as line length increases, thresholds improve steadily to approach the threshold plateau for the lowest blur level. Note, however, that for the largest blur level, we were unable to produce sufficiently long line lengths in order to expose a definitive plateau. Nevertheless, the data suggest that, provided line length is increased sufficiently, then thresholds become independent of blur level. The data suggest a process of ‘scale invariance’ in which stimuli which are magnified versions of one another (i.e. scaled in every respect–in this case both length and blur) produce identical levels of performance. This can be evaluated by replotting the data of Figure 2 on a scale invariant abscissa, namely line length divided by blur level. This is shown in Figure 3.

This procedure has the effect of collapsing together the data at different blur levels (although there remains a superiority of performance at the lowest blur level for observer GK). However, the overall success of this type of scaling indicates that any two stimuli of identical geometry (same ratio of line length to blur) will produce the same level of performance irrespective of the absolute size of the stimulus.

For each observer, the best-fitting bilinear fit to the log/log data is shown, with the constraint that the gradient of the linear fit at high ratios is zero (i.e. performance has reached a plateau). This plateau occurs just below 1° (0.90° for DW; 0.91° for DL; 0.73° for GK). The result of scaling the data in this way, and the subsequent bilinear curve fit accounts for 90% (DW), 89% (DL) and 89% (GK) of the total variance in the data. This indicates that the task of discriminating a deviation from linearity in a straight line is well described by a scale invariant mechanism, in which performance plateaus once line length exceeds approximately 40 times the level of blur of the line (the ‘knee’ point).

### Experiment 2

**Figure 4:**
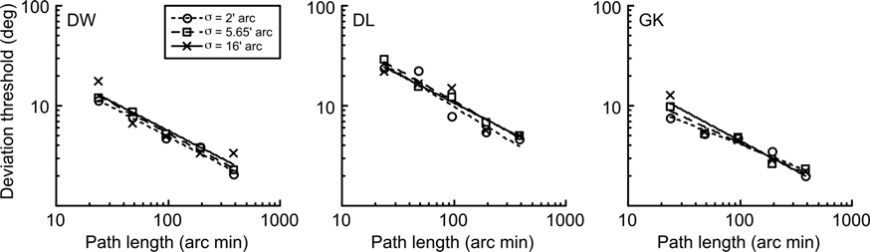
Path deviation thresholds for moving ‘blobs’. Thresholds for detecting a deviation in the path of a moving blob are plotted against path length. Individual panels show data for a single observer, and different symbols represent different levels of stimulus blur (σ = 2', circles; σ = 5.65', squares; σ = 16', crosses). Thresholds are considerably higher than those found for static line deviation judgments and fail to reach a plateau even at very long path lengths. Additionally, performance is not dependent on blob size: for any given path length thresholds are independent of blur. Data for each blur level are fitted by a power function whose exponent, averaged across blur levels and observers, is -0.58±0.07, indicating an approximate square root relationship between performance and path length. The data suggest that, for any given path length, some factor other than stimulus blur represents the limit to performance.

We next measured performance for discriminating path deviation for a moving blob as shown in Movie S1. Viewing distance was fixed whilst the size of blob was varied on the screen. Three blob sizes were investigated (σ = 2', 5.65' or 16'arc) whilst stimulus velocity was held constant at 3.5°/s. Path deviation thresholds for the three stimulus sizes are shown as a function of path length in Figure 4. A very different pattern of behavior emerges in comparison to the static line deviation judgments. Firstly, thresholds are considerably higher and fail to reach a plateau even at very long path lengths. Most significantly, performance exhibits no systematic dependence upon blob size–for any given path length thresholds are almost independent of blur. This represents the exact opposite trend to the scale invariant nature of static line deviation judgments (Figures 2 and 3) in which performance was directly proportional to the level of blur.

Thresholds are well described by a power function (straight line on log/log coordinates). The exponent of the power function, averaged across blur levels and observers, is −0.58±0.07, indicating an approximate square root relationship between performance and path length. One interpretation of these findings is that, for any given path length, some factor other than stimulus blur represents the limit to performance, hence the lack of effect of blur. The following experiment was designed to determine the nature of this limitation.

### Experiment 3

When objects are in motion in the real world, then variations in observer-object distance (as produced by self-motion towards the object) change the two-dimensional spatial characteristics of the object. If the viewing distance is halved, then for an object moving over a fixed physical distance, the object both doubles in size *and* the visual angle subtended by the motion path doubles. In addition, however, the angular velocity of motion doubles. The stimuli in Experiment 2 possessed a fixed velocity and so it could be argued that, whilst the stimuli accounted for scale invariance in the spatial domain (a range of stimulus sizes and path lengths were examined), the temporal aspects of scale invariance were ignored.

**Figure 5:**
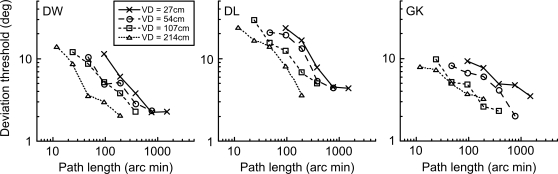
Path deviation thresholds for different viewing distances. Path deviation thresholds for moving blobs with a blur level of σ = 5.65' arc were measured at 4 viewing distances (VD), and are plotted against path length. Each panel shows data for a single observer and different symbols represent different distances. At all viewing distances, thresholds improve with increasing path length. At the shortest viewing distance (crosses), and hence largest size and highest velocity, longer path lengths are required to produce equivalent angular path deviation thresholds.

In this experiment we took this into account. The simplest way in which to produce a comprehensive set of scale invariant stimuli is to mimic what happens in the real world–maintain a number of stimuli of fixed size on the monitor and simply vary viewing distance. We therefore took the medium blur level stimulus series from Experiment 2 (square symbols in Figure 4) and examined path deviation thresholds at four different viewing distances. The results are shown in Figure 5.

**Figure 6:**
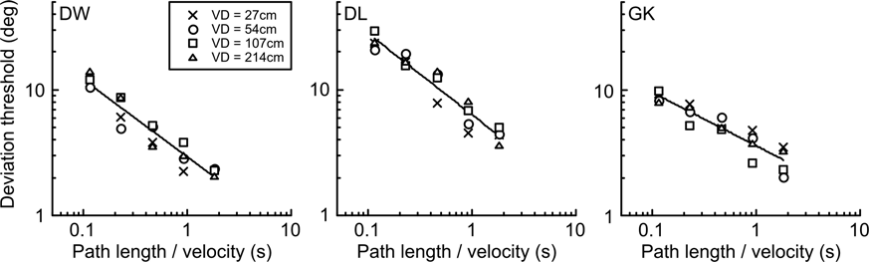
Relationship between path deviation thresholds and path duration. The path deviation thresholds from Figure 5 are replotted against an abscissa of path length/stimulus velocity. This ratio is equivalent to the duration of the motion path in seconds, and this has the effect of collapsing together data from different viewing distances. This indicates that, irrespective of the path length, stimulus blur or velocity of motion, path deviation thresholds depend upon the duration of the motion path in seconds. For each observer, the data are fitted with a power function, whose exponent, averaged across observers, is 0.56±0.12, indicating that deviation thresholds are approximately inversely proportional to the square root of path duration.

At all viewing distances, thresholds improve with increasing path length of motion. At the shortest viewing distance, and hence largest size and highest velocity (cross symbols), longer path lengths are required to produce equivalent angular path deviation thresholds. As in Figure 3 for the static line task, we could have collapsed the data by expressing path length as a proportion of stimulus size (blur). However, Experiment 2 tells us that stimulus blur is not the limiting factor to performance for discriminating deviation of these moving blobs. Instead, the higher stimulus velocity of the larger stimuli must be responsible for the variation in threshold at any given path length. This is taken into account in Figure 6 by expressing path length as a proportion of stimulus velocity. This proportion effectively reduces to the duration of the entire motion path in seconds, and has the effect of collapsing together data from different viewing distances.

Thus, irrespective of the path length, stimulus blur or velocity of motion, thresholds for discrimination of the angular deviation of a moving object depend upon the duration of the motion path in seconds. The data in Figure 6 are fitted with a power function, whose exponent, averaged across observers, is −0.56±0.12, indicating that deviation thresholds are approximately inversely proportional to the square root of path duration.

**Figure 7:**
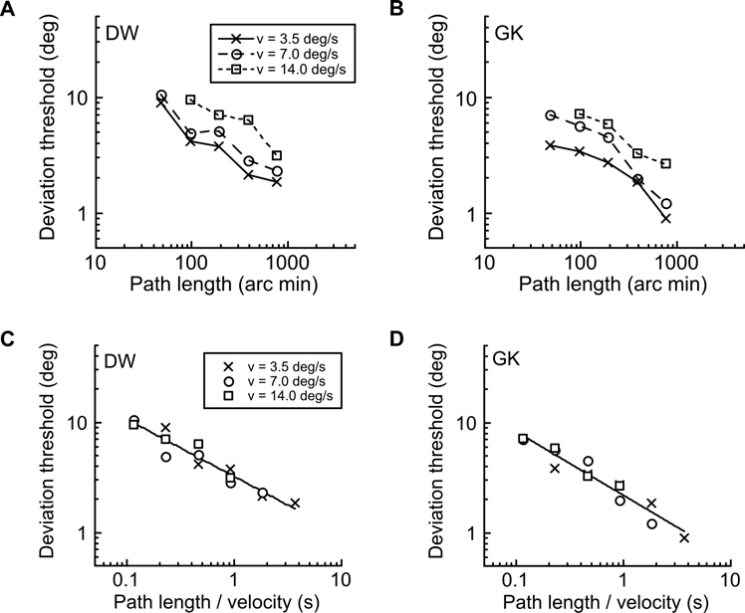
Path deviation thresholds for different stimulus velocities. Path deviation thresholds for moving blobs with a blur level of σ = 5.65' arc were measured at a viewing distance of 54 cm for 3 velocities (v). Panels A and B each show data for a single observer, plotted against path length, and different symbols represent different velocities. At all velocities, thresholds improve with increasing path length. As velocity increases, performance for any given path length is generally poorer. In panels C and D, the data from A and B are replotted against an abscissa of path length/stimulus velocity. This collapses together the data for different velocities and confirms that any two stimuli which are displayed for the same duration (same ratio of path length/velocity) will produce the same level of performance.

A control experiment was performed to ensure that this finding was not somehow an artifact of viewing distance. We therefore maintained viewing distance whilst varying the physical on-display velocity of a fixed-size blob. Figure 7 shows data for two of the observers. Panels A and B show that deviation thresholds are dependent upon velocity for a given path length but, when plotted against duration of motion path (panels C and D), thresholds for each velocity collapse together. Again, the results are consistent with a square-root relationship since the power function describing the relationship has an exponent of −0.51 (DW) and −0.58 (GK).

## Discussion

**Figure 8:**
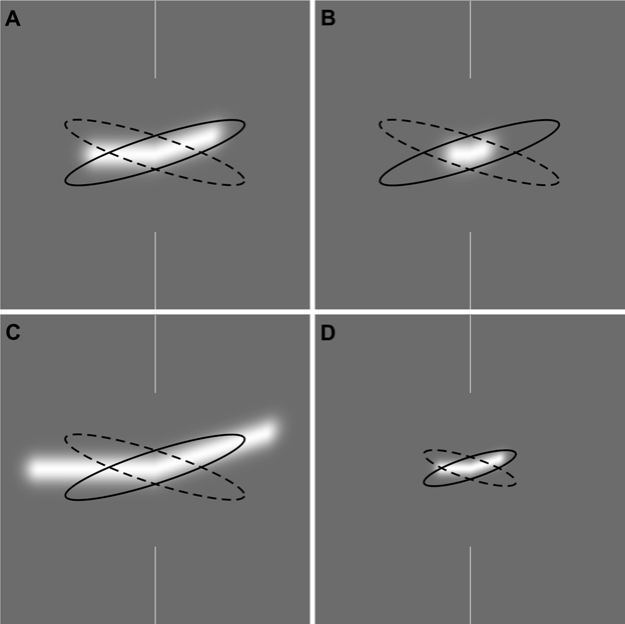
Modeling of angular discrimination performance for static lines. The results of Experiment 1 can be explained in the context of self-similar visual filters whose size is determined by stimulus blur. Panel A shows a receptive field arrangement which would produce a strong differential response to a deviation of a line dependent upon its direction of deviation. Filter size is determined by the stimulus blur. Shorter lines are clearly unsuited to produce the same differential response (B), whilst lines which extend beyond the filter result in no additional benefit (C). This explains the plateau in performance at very long line lengths (Figure 3). Spatially scaled versions of the filters result in the same level of performance (D).

Thresholds for the discrimination of an angular deviation within a static line stimulus improve with line length until reaching a plateau at just under 1°. Lines that possess the same ratio of length-to-blur result in the same angular deviation threshold. These results are straightforward to explain in the context of self-similar visual filters whose size is determined by stimulus blur (e.g. [12], [13]). For example, Figure 8A shows a receptive field arrangement which would produce a strong differential response to a deviation of a line dependent upon its direction of deviation. Shorter lines are clearly unsuited to produce the same differential response (Figure 8B), whilst lines which extend beyond the filter result in no additional benefit (Figure 8C), hence the plateau in performance at very long line lengths (Figure 3). Spatially scaled versions of the filters result in the same level of performance (Figure 8D). Scale invariance is a pervasive property of human vision, and holds for positional acuities [14], [15], visual illusions [16], [17] and texture discrimination [18]–[21], in addition to the angular judgments examined here.

The current data for static line deviation are remarkably similar to performance in both single line orientation and curvature discrimination tasks [22], [23]. Both orientation and curvature thresholds (when defined in angular terms) demonstrate scale invariance in that performance across a range of blur levels and line lengths collapse together as function of stimulus length divided by blur. The same trend is shown, with performance improving with line length before reaching a plateau. Optimum thresholds ranging from just over 1° [22] to just over 0.5° [23] are also similar to the optimum angular deviation thresholds found in the present study (Figure 3).

The path deviation thresholds for the moving stimuli are far more intriguing. Experiment 2 tells us that, whatever the mechanism involved, it is not tuned to the level of blur in the stimulus–a result which is diametrically opposite to that for the static line deviations of Experiment 1. This result, along with the relatively poor angular deviation thresholds (note that the scale of the y-axis in Figure 4 is an order of magnitude greater than in Figure 3), suggests that the analysis of path deviation for moving objects is determined by a large, fixed size filter which cares little about stimulus blur until this reaches extreme levels. Experiment 3 indicates that stimulus velocity is the critical factor determining path deviation thresholds at any given path length. A form of scale invariance is again exhibited (Figure 6), but this time in the temporal domain, reflecting the fact that the retinal velocity of a moving object varies with viewing distance. Once velocity is accounted for, we are left with the simple, if rather surprising, conclusion that path deviation thresholds depend solely upon the temporal duration of the motion path. Whilst deviation performance does improve with both longer paths and slower velocities (Figure 5), it does so only because both of these factors tend to produce longer durations of motion. When these two factors are combined to form path duration, data from a variety of different conditions collapse to form a single function, in which longer durations result in better performance.

What other evidence exists to suggest that path duration represents the determining factor in the analysis of path deviation? Sekuler et al. [24] measured simple reaction times (RTs) to large (30°) path deviations for objects moving at various velocities (2, 4 or 8°/s). They found that reaction times for different velocities collapsed together when expressed in terms of the duration of the pre-deviation path rather than its spatial extent. Their conclusion was that the visual system requires time to extract a sufficiently precise estimate of pre-deviation direction in order to react to a change in this direction. Sekuler et al. [24] present evidence to indicate that this form of global recruitment of direction continues across a relatively lengthy timescale of 500–700 ms pre-deviation duration. We present our data in terms of the total path duration (Figure 6), so our values need to be halved to correspond to pre-deviation duration, but our data confirm the view that estimates of motion direction continue to improve until 700 ms at least. Further support for this rather lengthy process comes from studies which have investigated the detection of a single dot moving along a consistent trajectory within random-direction dot noise [25], [26]. Detection performance is found to improve over durations extending to at least 600 ms [26] and is best described by a ‘cascade’ of motion detectors which, when activated, facilitate detectors tuned to a similar direction and whose receptive fields follow the path of the motion. Krekelberg and Lappe [27] also present evidence in terms of the positional misperception of moving dots to indicate that temporal recruitment along the trajectory of a moving object continues to at least 500 ms duration.

In terms of our path deviation task, we propose that the internal estimates of both pre- and post-deviation paths become more precise (less noisy) as duration increases, through an integration of directional information corresponding to the trajectory path. Contrary to the static condition, however, this is not a *spatial* integration of information as is known to improve line orientation judgments [28], [29]. Rather, it is a temporal integration of information which allows the observer to reconstruct an accurate measure of the direction of travel. The more time available, the better the estimate becomes, irrespective of the extent or velocity of travel alone. Local processing of motion direction is most likely obtained at various positions along the trajectory path by direction-selective simple cells in V1. At a later stage, the extracted motion energy samples are integrated across orientation and two-dimensional space in order to recover the global motion path. It is at this stage, presumably the middle temporal visual area (MT), where the directional quality of the global motion signal is enhanced by summation across time. The approximately square root relationship between performance and path duration is close to ideal, and suggests that this summation of independent time samples is very efficient.

Our results cannot be explained on the basis of temporal integration creating a static spatial ‘streak’ on which to base directional motion judgments [2]–[5], [30]. This is despite the fact that we used suitably high motion velocities [5]. Our reasoning is straightforward–that the behavior of discrimination thresholds for moving stimuli (Experiments 2 and 3) exhibit such a completely different pattern to that for static representations of the motion path (Experiment 1). This is inconsistent with a common spatial mechanism potentially afforded by motion ‘streaks’. Watamaniuk [31] also argues against a similar explanation for performance in a task where part of a dot's trajectory is occluded.

The motion paths which we used were all symmetric about the midline in terms of their duration and spatial extent. It would be interesting to vary the extents of the pre- and post-deviation paths independently to examine their relative importance in deviation discrimination. At the extremes, of course, both paths have the potential to limit performance. It is no good having a very accurate estimate of post-deviation path direction if pre-deviation direction is highly uncertain, and vice versa. This issue warrants further investigation.

In summary, path deviation discrimination for static lines and moving blobs involves completely different mechanisms. Performance for discriminating a deviation in a line is entirely consistent with well-established scale-invariant models incorporating spatial visual filters. Path deviation for moving blobs involves a global mechanism that recruits local motion detectors sharing common directional selectivity, whose activity defines the pre- and post-deviation path, and hence the direction of deviation. The limit to performance is determined neither by extent of motion path nor by velocity, but by the duration of the motion path.

## Materials and Methods

The three authors, all of whom had normal vision, participated in the experiments. DW and DL gathered data at Berkeley whilst observer GK gathered data at Bradford. The Berkeley stimuli were displayed on a Sony Multiscan G400 monitor with a Macintosh G4 as host computer. The corresponding apparatus at Bradford was an Apple LCD Cinema Display and a Macintosh G4. Stimuli were generated using the macro capabilities of NIH Image (v1.61) and were presented against a grey background of luminance 41 cd/m^2^. The standard viewing distance was 93 cm (Berkeley) or 107 cm (Bradford) in order to produce a common inter-pixel angular subtense of 1'arc. In Experiment 3, four different viewing distances were used (186 cm, 93 cm, 46 cm and 23 cm at Berkeley; 214 cm, 107 cm, 54 cm, 27 cm at Bradford).

The moving stimuli were Gaussian blobs of 0.99 Weber contrast whose standard deviation (σ) was varied in order to produce stimuli of different size. The blobs moved from left to right across the screen (Movie S1) until the midline (defined by two vertical lines 1'arc wide and 128' arc in length leaving a vertical gap of 256'arc between them). The initial path direction of the blobs was randomized from trial-to-trial with the constraint that this was within 35° either side of the horizontal. The vertical position of the blob at the horizontal midline was always mid-way between the two vertical reference lines. At this point the path direction of the blob could change, either deviating upwards or downwards, but maintaining the same linear velocity as pre-deviation. On any trial, one of seven equally spaced deviations could be presented: −3, −2, −1, 0, 1, 2 or 3 multiples of step size, where negative values indicate upward deviations, positive values represent downwards. These seven levels were randomly interleaved within a method of constant stimuli. After each trial, observers responded as to whether they thought the direction of deviation was upwards or downwards. Angular step size was chosen such that responses ranged from approximately 100% upwards to 100% downwards. Resulting data (from a minimum of 20 trials at each stimulus level) were fitted with a logistic function of the form:
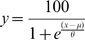
where *y* is the percentage of ‘upwards’ responses to a given deviation (*x*), *µ* is the deviation resulting in 50% upwards responses and *θ* is an estimate of threshold deviation (approximately half the distance between the 73% and 27% ‘upward’ response levels). Threshold deviations were established for a range of velocities, blob sizes (defined by the σ of the Gaussian luminance profile) and path lengths (the overall distance travelled in degrees of visual angle).

Static stimuli were also produced, representing the trace of the moving blob during its motion path. Essentially this produces a straight line with a ‘kink’ at the midpoint where the line either deviates upwards or downwards. The observers were simply asked to judge whether the line deviated upwards or downwards to the right of the horizontal midline. Again, a variety of blur (size) levels and path lengths were produced (obviously velocity is not a relevant parameter for these stimuli), and examples are provided in Figure 1 (A–D). Exposure duration for these static stimuli was fixed at 250 ms. Pilot data indicated that this was sufficiently long to allow observers to reach optimum performance under all conditions. In other words, thresholds are not limited by exposure duration and reflect the limits imposed by other factors such as size and path length.

## Supporting Information

Movie S1 An example of the moving stimulus. The blob moves from left to right before deviating at the midline. The observer is required to decide whether the deviation is upwards or downwards. For demonstration purposes, the deviation shown is grossly suprathreshold.
(0.10 MB MOV)
